# Impact of inducible nitric oxide synthase (iNOS) expression on triple negative breast cancer outcome and activation of EGFR and ERK signaling pathways

**DOI:** 10.18632/oncotarget.19631

**Published:** 2017-07-26

**Authors:** Pablo Garrido, Aliaa Shalaby, Elaine M. Walsh, Nessa Keane, Mark Webber, Maccon M. Keane, Francis J. Sullivan, Michael J. Kerin, Grace Callagy, Aideen E. Ryan, Sharon A. Glynn

**Affiliations:** ^1^ Discipline of Pathology, Lambe Institute for Translational Research, School of Medicine, National University of Ireland Galway, Galway, Republic of Ireland; ^2^ Medical Oncology, Galway University Hospital, Galway, Republic of Ireland; ^3^ Prostate Cancer Institute, National University of Ireland Galway, Galway, Republic of Ireland; ^4^ Discipline of Surgery, Lambe Institute for Translational Research, School of Medicine, National University of Ireland Galway, Galway, Republic of Ireland; ^5^ Discipline of Pharmacology and Therapeutics, Lambe Institute for Translational Research, School of Medicine, National University of Ireland Galway, Galway, Republic of Ireland; ^6^ Regenerative Medicine Institute (REMEDI), Biomedical Sciences, National University of Ireland Galway, Galway, Republic of Ireland; ^7^ Apoptosis Research Centre, National University of Ireland Galway, Galway, Republic of Ireland

**Keywords:** triple negative breast cancer, EGFR, inflammation, metastasis, nitric oxide

## Abstract

Inflammation is implicated in triple negative breast cancer (TNBC) progression. TNBC carries a worse prognosis than other breast cancer subtypes, and with the clinical and molecular heterogeneity of TNBC, there is a lack of effective therapeutic targets available. Identification of molecular targets for TNBC subtypes is crucial towards personalized patient stratification. Inducible nitric oxide synthase (iNOS) has been shown to induce p53 mutation accumulation, basal-like gene signature enrichment and transactivation of the epidermal growth factor receptor (EGFR) via s-nitrosylation. Herein we report that iNOS is associated with disease recurrence, distant metastasis and decreased breast cancer specific survival in 209 cases of TNBC. Employing TNBC cell lines representing normal basal breast, and basal-like 1 and basal-like 2 tumors, we demonstrate that nitric oxide (NO) induces EGFR-dependent ERK phosphorylation in basal-like TNBC cell lines. Moreover NO mediated cell migration and cell invasion was found to be dependent on EGFR and ERK activation particularly in basal-like 2 TBNC cells. This occurred in conjunction with NF-κB activation and increased secretion of pro-inflammatory cytokines IL-8, IL-1β and TNF-α. This provides substantial evidence for EGFR as a therapeutic target to be taken into consideration in the treatment of a specific subset of basal-like TNBC overexpressing iNOS.

## INTRODUCTION

Triple negative breast cancers (TNBC) account for 15-20% of breast cancers and are characterized by a lack of estrogen receptors (ER), progesterone receptors (PR) or HER2 receptors [1, 2]. TNBC patients have limited treatment options, exhibit earlier recurrence and poorer prognosis compared to non-TNBC patients [3]. TNBC is in reality a highly diverse group of cancers and despite the efforts of several groups in identification of phenotype/subtype specific expression patterns and gene signature-classification models, controversy remains regarding their subtyping [4, 5]. Nevertheless, most of the classifications agree in subdividing them into at least basal-like, mesenchymal, and luminal androgen receptor (LAR). Basal-like tumors constitute over 70% of the TNBC [4], and are further subdivided into basal-like 1 (BL1) and basal-like 2 (BL2). BL1 tumors are mainly driven by BRCA and cell cycle/cell division pathways, while the BL2 are mainly driven receptor tyrosine kinases (RTKs) [4, 6]. Improved stratification of this heterogeneous group is crucial to find new therapeutic strategies for TNBC.

Nitric oxide (NO) is a small biomolecule which exerts multiple effects on tumor biology [7, 8]. NO regulates a wide range of intracellular events through s-nitrosylation and regulation cell redox [9, 10]. The biological effects of NO are dose and temporal dependent with the ability to either inhibit or stimulate cell proliferation, migration and apoptosis [7, 11, 12]. Inducible nitric oxide synthase (iNOS) is a pro-inflammatory enzyme implicated in chronic inflammation and wound healing which synthesizes NO [13]. We previously demonstrated that iNOS is associated with poor survival in ER-negative breast cancer patients and enhanced expression of basal-like gene signatures [14]. Additionally we demonstrated that iNOS was associated with epidermal growth factor receptor (EGFR) activation [14] via s-nitrosylation [9]. This led us to hypothesize that iNOS may play a role in the progression of basal-like TNBC via enhanced activation of EGFR signaling. This study aims to identify the degree to which iNOS pays a role in TNBC progression and patient survival, in addition to identifying whether NO related activation of key signaling pathways that mediate cell migration and invasion are dependent on the EGFR.

Herein, we demonstrate that iNOS is associated with poor outcome in TNBC patients (n=209). We demonstrate that in BL2 TNBC, NO activation of the EGFR/ERK signaling pathway plays a direct role in the development of a pro-inflammatory phenotype that increases tumor cell invasive capacity. These results highlight a novel mechanism that may explain the observation that TNBC patients expressing iNOS display a worse prognosis. Additionally, these results highlight the potential of iNOS and EGFR as targets for consideration in the development of novel treatments for basal-like TNBC.

## RESULTS

### iNOS predicts poor survival in TNBC

**Figure 1:**
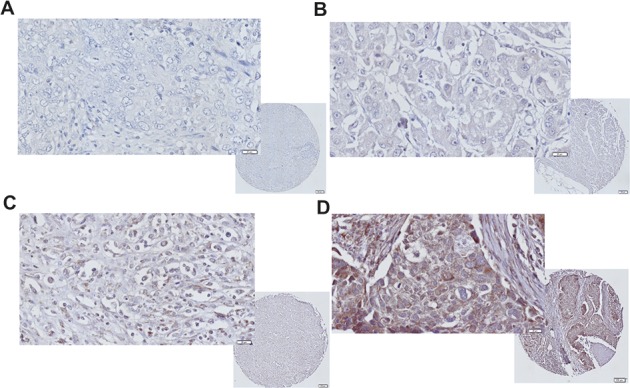
iNOS expression immunohistochemistry in TNBC Representative images of TNBC tumor with **(A)** negative, **(B)** low, **(C)** moderate, and **(D)** strong iNOS tumor epithelial staining.

**Figure 2:**
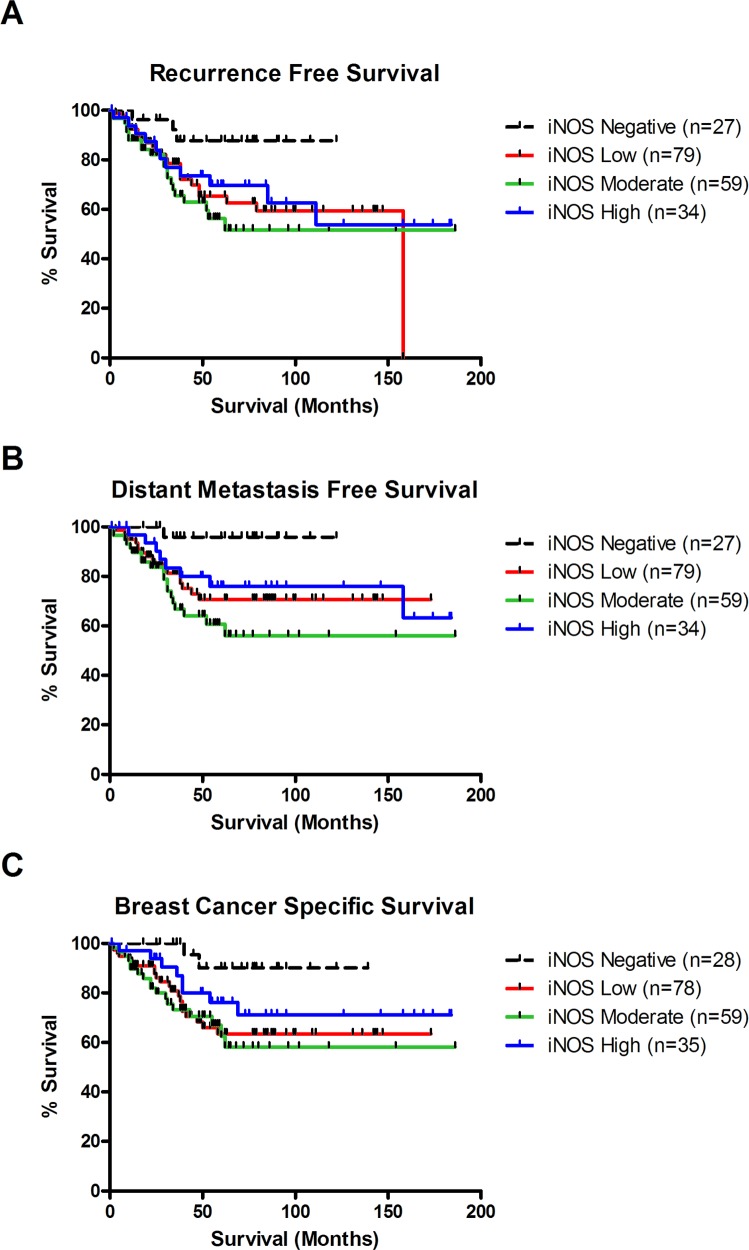
iNOS expression is associated with decreased recurrence free survival, distant metastasis free survival and breast cancer survival in TNBC **(A)** Kaplan-Meier cumulative recurrence free survival curves for TNBC patients by iNOS status (n = 199). Patients with weak iNOS expression (n = 79) or moderate iNOS expression (n = 59) were significantly associated with decreased recurrence free survival compared to patients with negative iNOS expression (n = 27) (P=0.036 & P=0.007, log-rank test). Patients with strong iNOS expression (n = 34) showed a trend toward worse survival compared to patients with negative iNOS expression (n = 27), but did not reach significance (P=0.078, log-rank test). **(B)** Kaplan-Meier cumulative distant metastasis free survival curves for TNBC patients by iNOS status (n = 199). Patients with weak iNOS expression (n = 79) or moderate levels of iNOS expression (n = 59) were significantly associated with increased risk of distant metastasis compared to patients with negative iNOS expression (n = 27) (P=0.017 & P=0.002, log-rank test). Patients with strong iNOS expression (n = 34) showed a trend toward increased risk of distant metastasis compared to patients with negative iNOS expression (n = 27), but did not reach significance (P=0.052, log-rank test). **(C)** Kaplan-Meier cumulative breast cancer–specific survival curves for TNBC patients by iNOS status (n = 200). Patients with weak iNOS expression (n = 78) or moderate levels of iNOS expression (n = 59) were significantly associated with increased risk of death due to breast cancer compared to patients with negative iNOS expression (n = 28) (P=0.012 & P=0.007, log-rank test). Patients with strong iNOS expression (n = 35) showed a trend toward increased risk of distant metastasis compared to patients with negative iNOS expression (n = 28), but did not reach significance (P=0.093, log-rank test).

We previously showed iNOS is a predictor of poor prognosis in a US cohort of ER-negative breast tumors but were unable to assess its role in TNBC [14]. Here we investigated the association of iNOS expression with recurrence free survival, metastasis free survival and breast cancer specific survival in 209 TNBC patients diagnosed in Ireland from 1999-2015 (Table 1 - Patient characteristics). TNBC tissue microarrays were stained for iNOS using immunohistochemistry and tumor epithelial iNOS expression assessed (Figure 1). As previously reported iNOS staining was predominantly observed in the tumor epithelia with few infiltrating immune cells staining positive for iNOS [14]. Recurrence free (local, regional or distant disease) survival analysis (Figure 2A) showed that iNOS positive tumors display decreased recurrence free survival compared to iNOS negative tumors, with low (Log-rank test: p=0.067) to moderate (p=0.010) levels of iNOS expression trending towards a significant association with disease recurrence. The highest levels of iNOS showed a trend towards decreased recurrence free survival but did not reach statistical significance (p=0.156). Metastasis free survival analysis (Figure 2B) showed that iNOS positive tumors have an increased chance of distant metastasis compared to iNOS negative tumors, with low (Log-rank test: p=0.029) to moderate (p=0.002) levels of iNOS expression significantly associated with the development of distant metastasis. Similar to disease-free survival, the highest levels of iNOS showed a trend towards increased metastasis but did not quite reach statistical significance (p=0.065). Given that recurrence whether local, regional or distant is suggestive of aggressive progressive disease we then examined whether NOS2 predicts increased risk of dying from TNBC. Similarly when examining breast cancer specific survival, Figure 2C showed that iNOS positive tumors are associated with worse breast cancer specific survival compared to iNOS negative tumors, with low (Log-rank test: p=0.020) to moderate (p=0.006) levels of iNOS expression significantly associated with decreased survival. The highest levels of iNOS showed a trend towards decreased survival but did not reach statistical significance (p=0.189). The lack of association with survival at the highest levels of iNOS may reflect the generation of NO levels that begin to switch from pro-tumorigenic effects to anti-tumorigenic effects as previously reviewed [7]. Cox regression survival analysis was performed in Table 2 to calculate the hazard ratios (HR) associated with iNOS levels and recurrence free survival, metastasis free survival and breast cancer specific free survival. Most notably low levels of iNOS were associated a breast cancer specific free survival HR of 4.8 (95% Confidence Interval (CI) 1.11-20.41; p=0.035), while intermediate levels are associated with a HR of 5.9 (95% CI 1.37-25.64; p=0.017). iNOS remained a significant predictor of outcome in the multivariable analysis after adjustment for age at diagnosis, neoadjuvant therapy, tumor grade and histological subtype, with low levels of iNOS associated a breast cancer specific free survival HR of 5.0 (95% Confidence Interval (CI) 1.16-21.94; p=0.031), while intermediate levels are associated with a HR of 7.2 (95% CI 1.60-32.34; p=0.010) in the multivariable analysis (Table 2). Additionally we found that the percentage of tumor infiltrating lymphocytes (TILs) in the tumor adjacent stroma was highest in patients with iNOS negative tumors (Median % TIL count = 17.5%), and lowest in patients with iNOS moderate tumors (Median % TIL count = 10%) (Table 3). TIL counts increased again in patients with iNOS high tumors (Median % TIL count = 15%). These results warrant further investigation as the literature suggests that low TIL counts are associated with worse outcomes in TNBC [15].

**Table 1:** TNBC patient characteristics

Characteristic	Median (Range)
Age at Diagnosis (Years) (n=209)	57 (30-91)
Follow-Up (Months)	
Recurrence Free Survival (n=199)	38 (1-186)
Metastasis Free Survival (n=200)	40 (1-186)
Breast Cancer Specific Survival (n=200)	46 (1-186)
	**N (%)**
Tumor Grade	
Grade 2	27 (13%)
Grade 3	181 (87%)
Unknown	1
Histological Type	
Ductal	171 (82%)
Lobular	9 (4%)
Metaplastic	11 (5%)
Mixed ductal	2 (1%)
Papillary	1 (<0.5%)
Medullary	7 (3%)
Apocrine	6 (3%)
Micropapillary	1 (<0.5%)
Unknown	1 (<0.5%)
Neoadjuvant Therapy	
No	183 (88%)
Yes	26 (12%)
iNOS Levels	
Negative	28 (13%)
Weak	83 (40%)
Moderate	62 (30%)
Strong	36 (17%)
Disease Free Survival*	
No recurrence	140 (70%)
Recurrence	59 (30%)
Metastasis Free Survival*	
No distant metastasis	154 (77%)
Distant Metastasis	46 (23%)
Breast Cancer Specific Survival**	
Alive	151 (75%)
Deceased	49 (25%)

*9 patients excluded due to distant metastasis at diagnosis, and 1 treated in another country and lost to follow-up.

**9 patients excluded due to death from other causes.

**Table 2:** Association of iNOS with TNBC outcomes

	Univariate Cox Regression Analysis			
	HR	95% Confidence Interval	*P*-value	
iNOS Levels & Disease Free Survival*				
Negative	1.0			27
Low	3.5	1.04-11.53	0.042	79
Moderate	4.4	1.31-14.74	0.017	59
High	3.0	0.82-10.75	0.095	34
iNOS Levels & Metastasis Free Survival*				
Negative	1.0			27
Low	8.0	1.06-59.75	0.043	79
Moderate	12.1	1.61-90.06	0.015	59
High	6.5	0.80-51.89	0.080	34
iNOS Levels & Breast Cancer Specific Survival				
Negative	1.0			28
Low	5.2	1.21-22.15	0.026	78
Moderate	6.0	1.38-25.70	0.017	59
High	3.5	0.74-16.55	0.112	35
	Multivariable Cox Regression Analysis**			
	**HR**	**95% Confidence Interval**	***P*-value**	**N**
iNOS Levels & Disease Free Survival				
Negative	1.0			27
Low	3.5	1.04-11.51	0.044	79
Moderate	4.6	1.35-15.79	0.015	59
High	2.8	0.75-10.12	0.127	33
iNOS Levels & Metastasis Free Survival				
Negative	1.0			27
Low	7.9	1.05-59.49	0.044	79
Moderate	12.7	1.67-95.91	0.014	59
High	6.1	0.75-49.76	0.091	34
iNOS Levels & Breast Cancer Specific Survival				
Negative	1.0			28
Low	5.3	1.24-22.90	0.024	78
Moderate	6.2	1.43-27.13	0.026	59
High	3.2	0.67-15.15	0.142	34

*Adjusted for age at diagnosis, no chemotherapy/neoadjuvant/adjuvant chemotherapy/unknown, tumor grade, histological subtype.

**Table 3:** Association between iNOS levels and % sTILs

iNOS Levels	Median % sTILS	Range	P value*	N
Negative	17.5	5-90	Ref	27
Low	15.0	2-80	0.272	79
Moderate	10.0	0-80	0.038	59
High	15.0	2-80	0.177	34

*Kwallis non-parametric test comparing sTILs levels in iNOS low, moderate and high tumors to iNOS negative tumors.

### NO activates EGFR signaling

**Figure 3:**
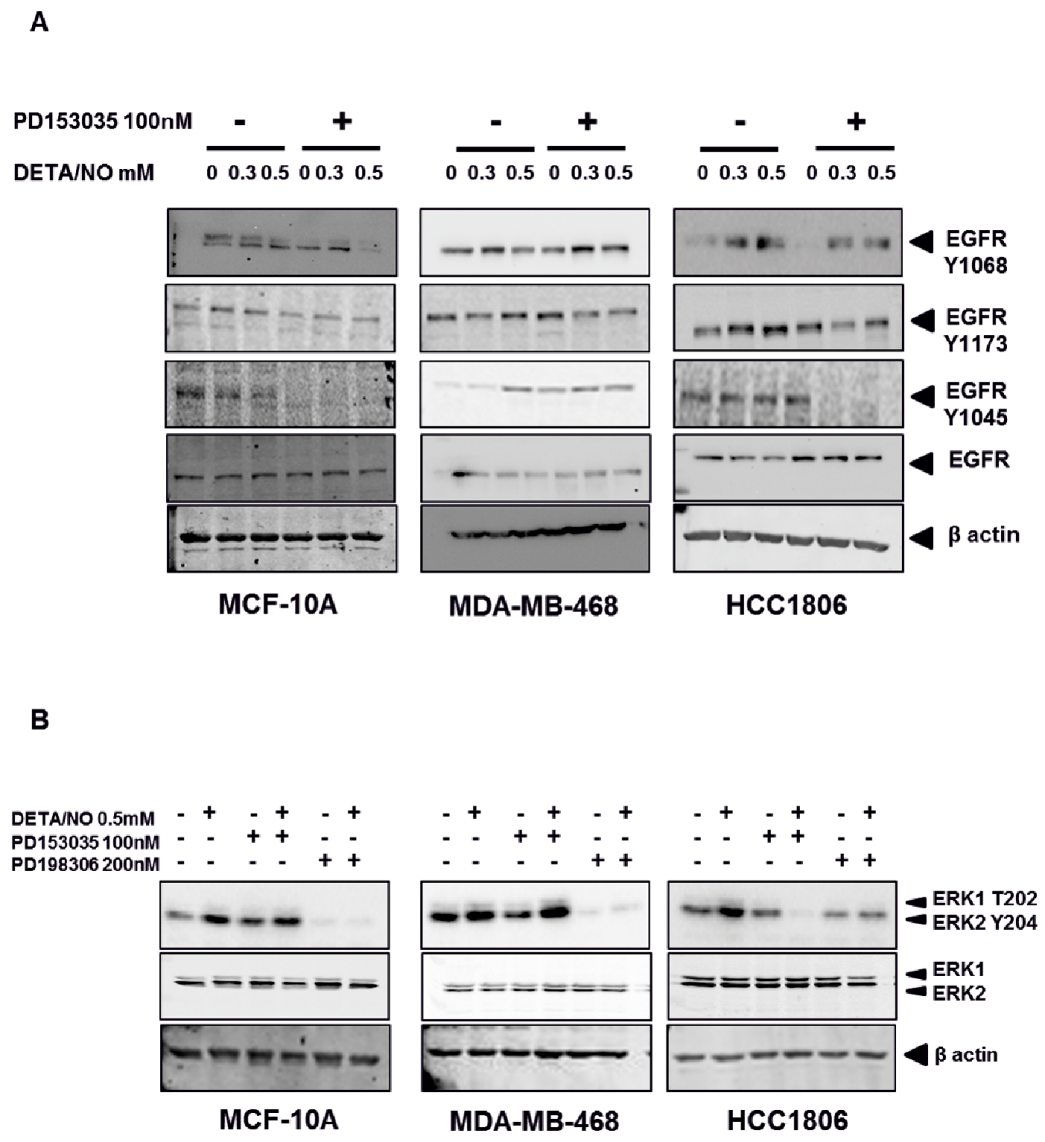
NO induces increased EGFR and ERK phosphorylation in TNBC cell lines Phosphorylation status of EGFR **(A)** in the MCF-10A, MDA-MB-468 and HCC1806 cell lines after 24 hours exposure to increasing doses of DETA/NO alone or in combination with 100nM of PD153035 (EGFR inhibitor). **(B)** Phosphorylation status of the MAP kinases ERK1 and ERK2 after 24 hours exposure to 0.5mM of DETA/NO alone or in combination with 100nM of PD153035 or 200nM PD198306 (MEK inhibitor).

**Figure 4:**
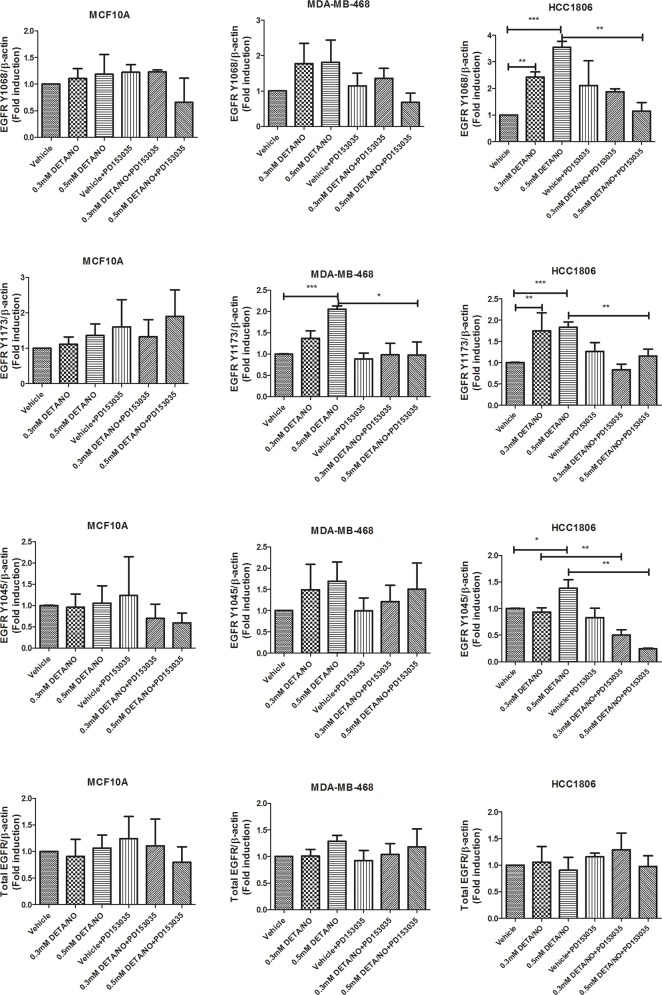
Densitometry analysis of EGFR phosphorylation in response to DETA/NO Quantification of western blots (Figure 3A) examining EGFR phosphorylation at Y1068, Y1173 and Y1045 and total EGFR expression in the MCF-10A, MDA-MB-468 and HCC1806 cell lines after 24 hours exposure to increasing doses of DETA/NO alone or in combination with 100nM of PD153035 (EGFR inhibitor).

To investigate the impact of NO on basal-like cancer cells, we used three cell lines: MCF-10A normal immortalized basal breast cells, MDA-MB-468 BL1 TNBC cells and HCC1806 BL2 TNBC cells according to Lehmann's categories [4, 6]. The basal expression levels of total EGFR and EGFR phosphorylation at Y1045, Y1068 and Y1173 are shown in Supplementary Figure 1A, while the basal levels of iNOS in these cell lines are show in Supplementary Figure 1B. We used increasing concentrations of the NO donor diethylenetriamine/nitric oxide adduct (DETA/NO), to assess the effects of NO on EGFR signaling, a marker of poor prognosis in basal-like TNBC [16, 17]. The levels of DETA/NO used in this study are equivalent to 0 to 500nM NO, which are physiologically relevant as it is estimated that approximately 100nM NO is required for the phosphorylation of Akt and 400nM for the phosphorylation of p53 *in vitro* [18, 19]. The effects of DETA/NO observed in this study are unlikely to be mediated by the NO-cGMP axis, as cGMP is activated at levels equivalent to <0.1mM DETA/NO [18, 19], and 0.5mM DETA/NO has been shown to suppress cGMP back to baseline levels [20]. Although NO has been shown to increase the phosphorylation status of EGFR residues [14], the downstream signaling effects remain unknown. We combined DETA/NO with the EGFR kinase inhibitor (PD153035) to study the reliance of NO on EGFR signaling. Phosphorylation on EGFR residues Y1045, Y1068 and Y1173 are recognized as being responsible for controlling EGFR signaling [21]. Figure 3A and the corresponding densitometry analysis in Figure 4 demonstrates that 24 hour exposure to DETA/NO increased EGFR phosphorylation in Y1173 in MDA-MB-468 and Y1045, Y1068 and Y1173 in HCC1806 cell lines. No effect was seen in MCF-10A. Interestingly the BL2 cell line, HCC1806 cell line, shows a higher induction of EGFR phosphorylation compared the BL1 cell line MDA-MB-468, with the 0.5mM dose of DETA/NO showing the strongest affect. The increased phosphorylation was reverted to basal levels when the DETA/NO treatment is combined with 100nM of PD153035 in the HCC1806 for Y1045, Y1068 and Y1173 and in MDA-MB-468 for Y1173. Interestingly NO treatment also increased the expression of iNOS mRNA which was reversed with the addition of the EGFR Inhibitor PD153035 indicating a feed forward loop via the EGFR (Supplementary Figure 1C).

**Figure 5:**
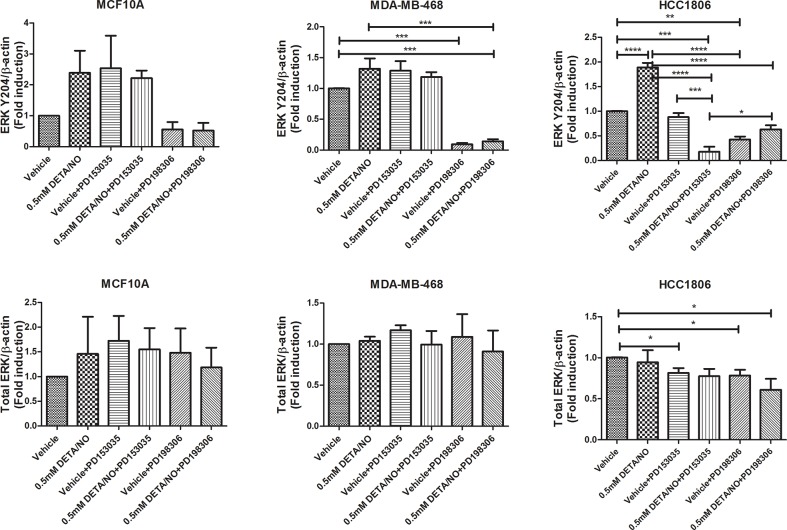
Densitometry analysis of ERK phosphorylation in response to DETA/NO Quantification of western blots (Figure 3B) examining ERK phosphorylation at Y204 and total ERK expression in the MCF-10A, MDA-MB-468 and HCC1806 cell lines after 24 hours exposure to increasing doses of DETA/NO alone or in combination with 100nM of PD153035 (EGFR inhibitor) or 200nM PD198306 (MEK inhibitor).

We next examined the effect of DETA/NO induced EGFR phosphorylation on ERK1/2 as one of its main downstream effectors. While 0.5mM of DETA/NO increased ERK1/2 activation in all cell lines, it was only statistically significant in HCC1806 as shown by the densitometry analysis in Figure 5. DETA/NO induction of ERK1/2 activation was reverted to below basal levels by the PD153035 only in the HCC1806 showing the specific EGFR-dependency of NO induction of ERK phosphorylation in this cell line (Figure 3B). ERK1/2 phosphorylation induced by DETA/NO was abrogated by combining the treatment with 200nM of MEK inhibitor PD198306 [22]. PD198306 showed significant activity in MDA-MB-468 and HCC1806, reducing ERK1/2 phosphorylation status in both basal and DETA/NO stimulated cells, indicating that NO activation of ERK is enhanced through MEK.

### EGFR activation by increased NO triggers a pro-inflammatory phenotype

**Figure 6:**
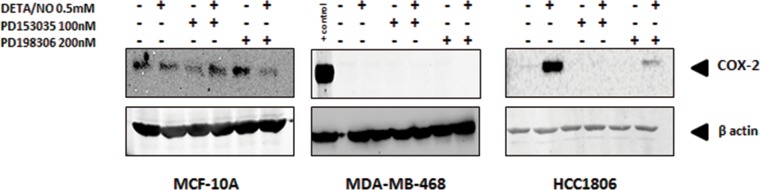
Induction of COX-2 by NO in HCC1806 is suppressed by EGFR or MEK inhibitiors COX-2 protein levels in MCF-10A, MDA-MB-468 and HCC1806 after 24 hours exposure to 0.5mM of DETA/NO alone or in combination with 100nM of PD153035 or 200nM PD198306.

**Figure 7:**
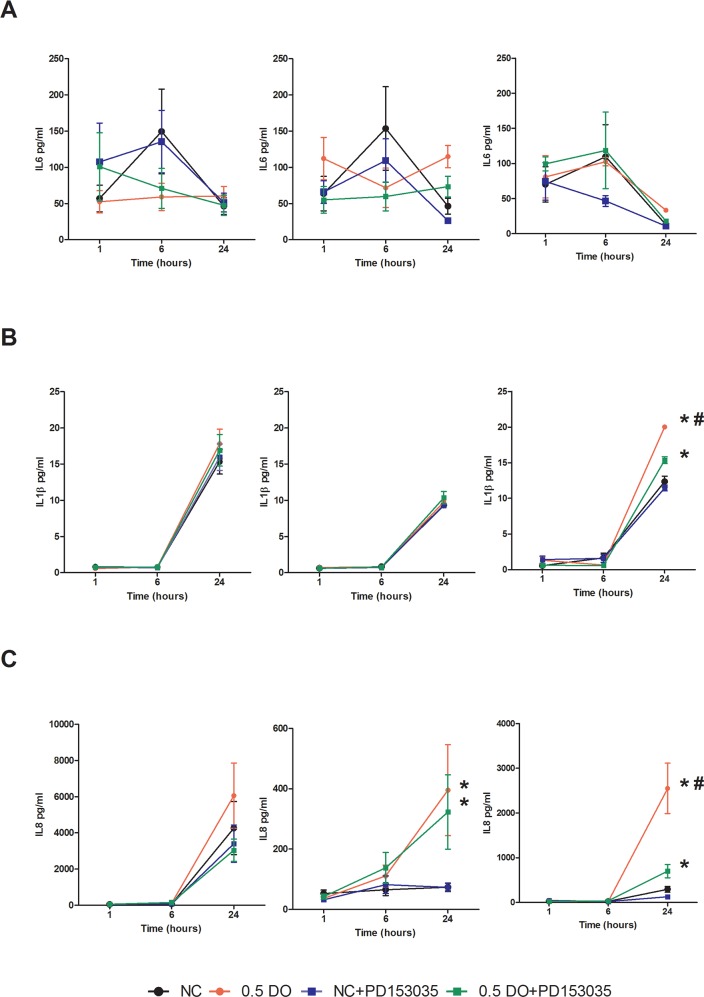
NO induction of IL-1β and IL-8 secretion is perturbed by EGFR inhibition in HCC1806 Cytokine profiling of released IL-6 **(A)** IL-1β **(B)** and Il-8 **(C)** in MCF-10A, MDA-MB-468 and HCC1806 after 24 hours exposure to vehicle or 0.5mM of DETA/NO alone or in combination with 100nM of PD153035. * p<0.05 vs vehicle; # p<0.05 vs DETA/NO plus PD153035

NO is largely recognized as a pro-inflammatory biomolecule [23], and is implicated in the establishment of the pro-inflammatory phenotype [24]. To further investigate the interaction between NO and EGFR in basal-like breast cancer, we first examined the effect of DETA/NO on cyclooxygenase-2 (COX-2) expression, which is implicated in tumor progression and poor outcome in ER negative breast cancer [25–27]. 0.5mM DETA/NO induced COX-2 expression in the BL2 HCC1806, which was significantly abrogated by EGFR and MEK/ERK1/2 inhibition, confirming the role of EGFR in COX2 induction (Figure 6). DETA/NO had no significant effect on COX-2 expression in the normal immortalized MCF-10A. COX-2 was not detectable in the BL1 MDA-MB-468 either at basal line or after DETA/NO treatment. Interleukin (IL)-1β, IL-6, IL-8 and tumor necrosis factor alpha (TNFα) are four cytokines involved in inflammation and wound healing [28–30]. We measured the levels of IL-1β, IL-6 and IL-8 secretion 1, 6 and 24 hours post treatment with 0.5mM DETA/NO alone or in combination with 100nM PD153035. Figure 7 shows there is no consistent secretion pattern applicable for the three different cytokines. No effect of DETA/NO treatment on IL-6 expression was found in any cell lines (Figure 7A). Figure 7B shows IL-1β secretion was significantly increased in HCC1806 24 hours post exposure to DETA/NO, and could be partially reversed with the EGFR inhibitor. DETA/NO increase IL-1β secretion from MCF-10A or MDA-MB-468. Figure 7C demonstrates no stimulatory effect of DETA/NO on MCF-10A secretion of IL-8; while both MDA-MB-468 and the HCC1806, show increased IL-8 secretion 24 hours post DETA/NO exposure compared to vehicle.

**Figure 8:**
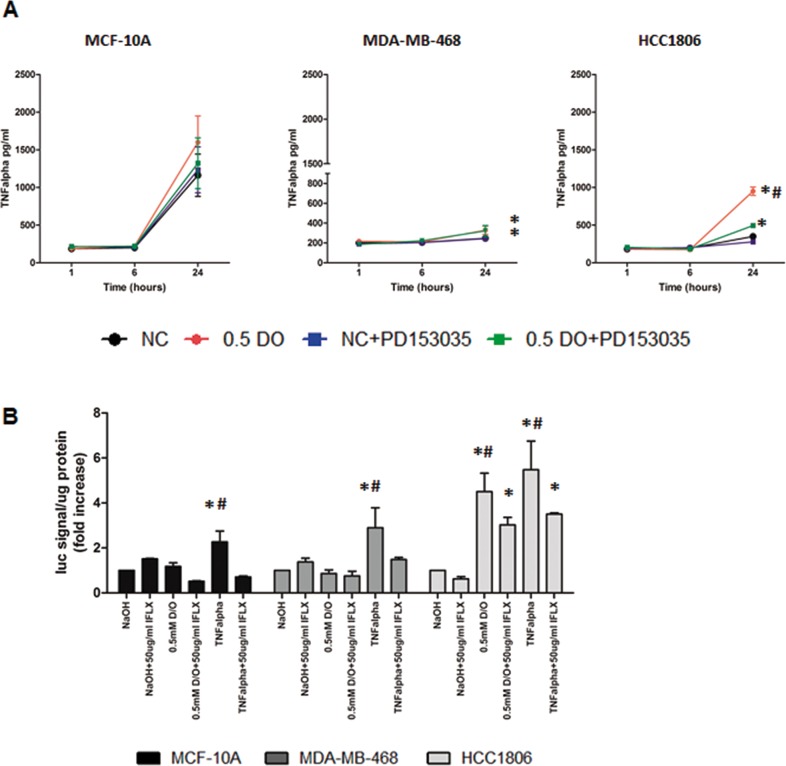
NO induces increased TNFα secretion and activation of NF-κB in HCC1806 TNFα released **(A)** in the three cell lines after 24 hours exposure to vehicle or 0.5mM of DETA/NO alone or in combination with 100nM of PD153035. **(B)** Luciferase activity of NF-κB reporter after 24 hours exposure to vehicle or 0.5mM of DETA/NO alone or in combination with 50μg/ml of Infliximab (TNFα blocking antibody), 10ng/ml of recombinant TNFα were used as a positive control. * p<0.05 vs vehicle; # p<0.05 vs DETA/NO plus PD153035.

TNFα plays a central role in inflammation in cancer. Figure 8A demonstrates that TNFα secretion is significantly increased by 0.5 mM DETA/NO compared to vehicle after 24 hours in both BL1 MDA-MB-468 and BL2 HCC1806 cells, but not in MCF-10A. The magnitude of TNFα induction in the HCC1806 was greater than the MDA-MB-468. Co-treatment with the EGFR inhibitor, significantly decreased TNFα release to basal levels in HCC1806 but not MDA-MB-468. This finding suggests a link between EGFR activation and the induction of a pro-inflammatory phenotype in the BL2 HCC1806. Nuclear factor-kappa B (NF-κB) meditates TNFα signaling. When TNFα binds to its cell surface receptor, it triggers intracellular signaling resulting in the translocation of NF-κB to the nucleus and the activation of NF-κB dependent gene expression [31]. We used a NF-κB luciferase reporter to study the effect of NO levels on NF-κB gene promoter dependent activity (Figure 8B). We show increased NF-κB activity after DETA/NO exposure in the BL2 HCC1806 cell line, while we could see no significant effect in neither the MCF-10A nor the BL1 MDA-MB-468 cells, which reflects the higher levels of TNF α induced by DETA/NO in Figure 8A. To confirm a direct TNFα mediated effect, we used the TNFα blocking antibody Infliximab to investigate the effect of the NO induced TNFα on NF-κB activity. Our findings indicate a significant decrease in NF-κB induced activity in the HCC1806 when we combined Infliximab with DETA/NO. Together, these findings suggest a direct role for NO, triggering a pro-inflammatory phenotype in the BL2 cells, mediated by EGFR and NF-κB activation.

### NO regulates migration and invasion capability

**Figure 9:**
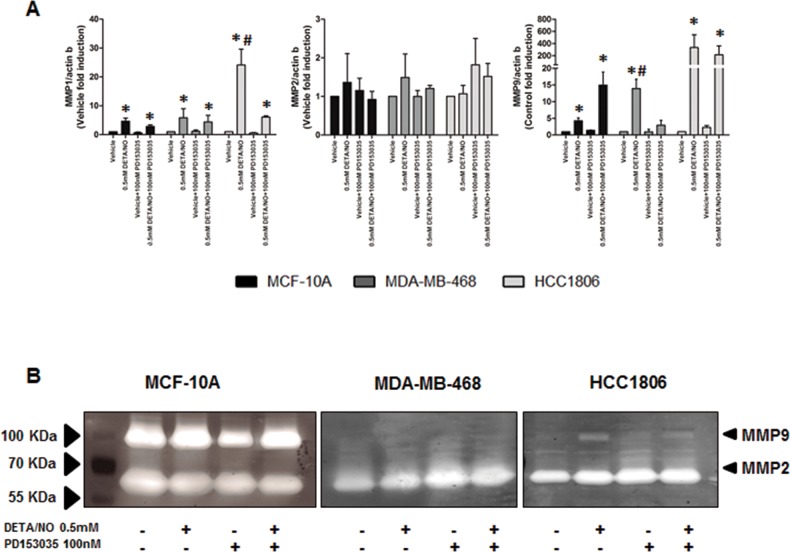
NO induces increased MMP expression and activity **(A)** qPCR showing the mRNA expression levels of MMP1, MMP2 and MMP9 after 24 hours exposure to vehicle or 0.5mM of DETA/NO alone or in combination with 100nM of PD153035. **(B)** Zymography showing the gelatinase activity of MMP9 and MMP2 after 24 hours exposure to vehicle or 0.5mM of DETA/NO alone or in combination with 100nM of PD153035.

Inflammation is a major driving force in cancer metastasis [32, 33]. IL-8 is a pro-inflammatory cytokine which induces matrix metalloproteinase (MMP9) expression and cleavage in different biological systems [34, 35]. To determine if the induction of IL-8 secretion in MDA-MB-468 and HCC1806 by DETA/NO as seen in Figure 7C, reflects the invasive potential of the TNBC cell lines we examined the effects of DETA/NO on MMP expression, and migration and invasion potential. 0.5mM DETA/NO increased levels of MMP1 and MMP9, but not MMP2 mRNA in all three cells lines (Figure 9A). To confirm that changes in gene expression translated to its biological activity we assessed DETA/NOs effect on MMP2 and MMP9 activity by gelatin zymography (Figure 9B). Surprisingly, the MCF-10A exhibited higher basal MMP activity than the cancer cell lines, with no significant effect of 0.5mM DETA/NO treatment on gelatinase activity. HCC1806 showed increased MMP9 activity after 24 hours exposure to DETA/NO, which was partially reversed in combination with the EGFR antagonist. DETA/NO had no significant effect on gelatinase activity in the MDA-MB-468.

**Figure 10:**
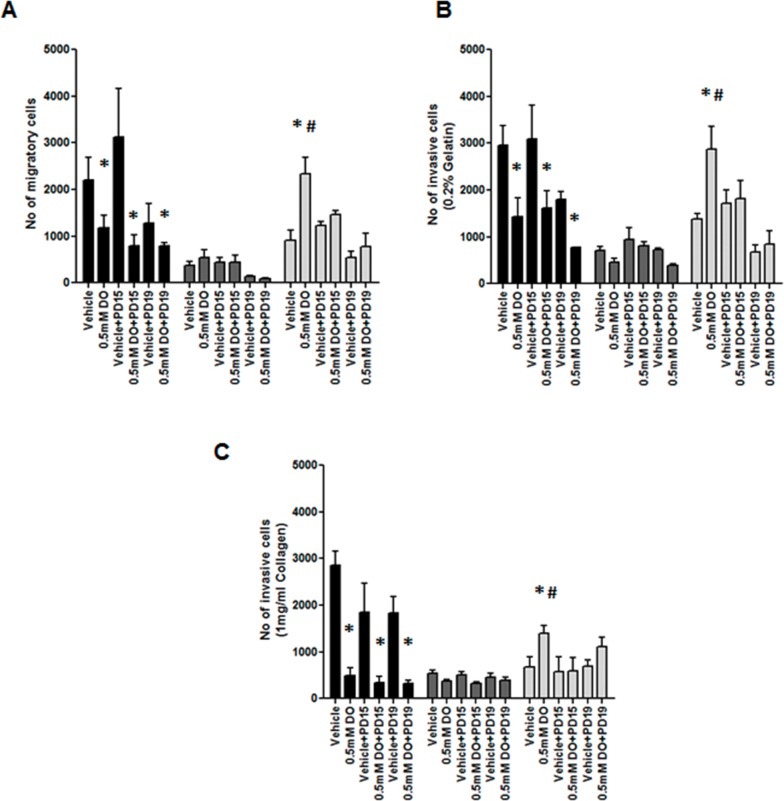
Impact of EGFR inhibition on NO induction of cell migration and invasion **(A)** Cell migration, or invasion through **(B)** gelatin and **(C)** collagen after 24 hours exposure to vehicle or 0.5mM of DETA/NO alone or in combination with 100nM of PD153035 or 200nM PD198306.* p<0.05 vs vehicle; # p<0.05 vs DETA/NO plus PD153035.

**Figure 11:**
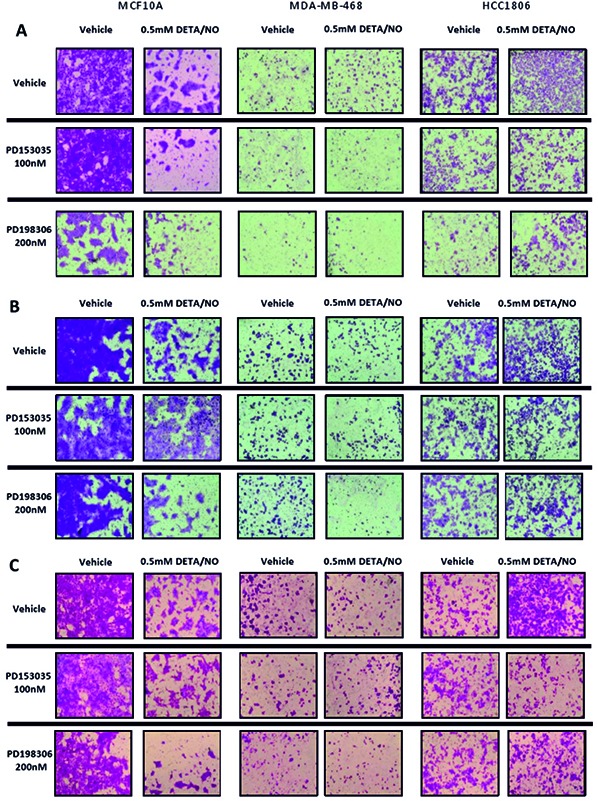
Representative images of NO effects on migration and invasion Representative images of migratory and invasion potential **(A)** and invasive potential of the cell lines to invade through a 0.2% gelatin **(B)** or 1mg/ml collagen **(C)** of the cell lines after 24 hours exposure to vehicle or 0.5mM of DETA/NO alone or in combination with 100nM of PD153035 or 200nM PD198306.

Tumor cells undergo migration and invasion during metastasis [36]. These mechanisms are considered identical to those exerted by non-neoplastic cells in physiological processes such as wound healing [37]. We examined the effect of 0.5mM DETA/NO on cellular migration and invasion capacity across a FBS gradient (5 to 10%) through 8μm trans-wells either uncoated (migration) or coated (invasion) (Figure 10 and Figure 11 (representative images)). 0.5mM DETA/NO significantly decreased the migration potential of normal MCF-10As, and conversely increased migration in both basal-like TNBC cell lines, with a greater increase seen in BL2 HCC1806 compared to BL1 MDA-MB-468. DETA/NO mediated migration was dependent on the EGFR/ERK activation, as either EGFR (PD153035) or MEK (PD198306) inhibition reverted migration levels to baseline. Similar patterns were observed for invasive capacity (Figure 9A). DETA/NO also increased the rate of HCC1806 invasion through gelatin (Figure 9B), and collagen coated surfaces (Figure 9C) confirming the effect observed on MMP-9 activity by zymography, and also indicating that DETA/NO may increase MMP1 enzymatic activity in addition to mRNA levels. In contrast, the MDA-MB-468 cells do not show any difference in the invasive capability after DETA/NO exposure, and the MCF-10A show a reduced invasive capacity similar to their migration capacity. Neither the EGFR nor the MEK inhibitors had any significant effect on MCF-10A or MDA-MB-468 invasive potential. These results reiterate, the dependency of BL2 TNBC cells on growth factor signaling, making them more sensitive to NO-related effects. These findings support the idea that high NO levels, produced by increased iNOS expression, trigger a loop where EGFR-ERK and NF-κB are the main effectors, and implicate iNOS in TNBC progression.

## DISCUSSION

Finding new targets for the treatment of TNBC is of major clinical importance. TNBCs have a high risk of local and distant recurrence, particularly in the first three years post diagnosis. TNBCs account for 15-20% of all breast cancers and are associated with an earlier onset, BRCA1 mutations and patients of African descent [38]. Current treatment strategies involve surgical intervention coupled with standard chemotherapy (anthracyclines, taxanes, cyclophosphamide), in the neoadjuvant or adjuvant setting. Recent clinical trials have focused around platinum agents and PARP inhibitors (particularly in BRCA1 associated TNBC), androgen receptor antagonists (LAR TNBC) and immune checkpoint inhibitors (TNBCs with high levels of tumor infiltrating lymphocytes) [38].

We previously showed that iNOS is a predictor of poor survival in ER-negative breast cancer (n=98), but not in ER-positive breast cancer in a cohort of US patients [14]. We further demonstrated that iNOS was associated with EGFR phosphorylation, basal-like transcription patterns, and predicted poor outcome in patients with basal-like breast cancer (n=41) [14]. Subsequently, we showed that NO autophosphorylates the EGFR via S-nitrosylation [9]. In 2015 Granados-Principal et al demonstrated an association between high levels of iNOS and decreased relapse free survival in 83 cases of TNBC [39]. Moreover in 2014, Heinecke et al demonstrated that TNBC tumor proliferation and metastases to the brain were significantly suppressed by iNOS inhibition in nude mice [23], recapitulated by Granados-Principal et al [39]. Herein we demonstrate in an Irish cohort of 209 TNBC patients that iNOS predicts decreased overall survival, which includes patients with basal-like TNBC (n=136), corroborating our previous findings and validating iNOS as a predictor of poor outcome in patients from different geographic regions (USA and Ireland).

With Lehmann et al's identification of six TNBC molecular subtypes (BL1, BL2, immunomodulatory, mesenchymal, mesenchymal stem like and LAR) based on gene expression profiles [4], and their further refinement into four TNBC molecular subtypes (BL1, BL2, mesenchymal, and LAR) [6], it becomes apparent that there are two different basal-like tumor biology's at play. Within the basal-like tumors, BL2 TNBC are characterized by a greater dependency on growth factor signaling including the EGFR, as opposed to BL1 where the tumors are mainly driven by cell cycle genes and BRCA mutations [4]. Given this further subdivision of the basal-like phenotype we examined whether increased levels of NO influence basal-like breast cancer cells, and regulate their pro-inflammatory and metastatic capacities in the context of BL1 and BL2 phenotypes. While both cell lines representing the BL1 and BL2 subtypes, displayed increased EGFR phosphorylation in response to DETA/NO not only in the previously reported Y1045 and Y1173 residues [14], but also in Y1068, the effect was greater on the BL2 cell line HCC1806. It should be noted that the induction of Y1045 was modest at best which may reflect our previous observation that Y1045 was phosphorylated within 10 mins exposure to DETA/NO [14], and therefore there may be some degree of a negative feedback loop at 24 hours post incubation. The activation of the Y1068 by DETA/NO directly links NO mediated EGFR activation with ERK/MAPK activation [21], which we demonstrated was abrogated by the inclusion of the MEK/ERK inhibitor PD198306 [40]. This induction of ERK activation was dependent on the EGFR in the BL2 HCC1806 but not the BL1 MDA-MB-468 as demonstrated by the effects of EGFR inhibition on ERK phosphorylation after DETA/NO exposure, supporting the reported dependency of the BL2 subtype on growth factor receptor signaling [4]. It should be noted however that the MEK inhibitor did repress ERK phosphorylation below base line (to approx. 50% of baseline) in the HCC1806, and while DETA/NO did increase ERK phosphorylation in the presence of the MEK inhibitor, it was unable to fully return ERK phosphorylation back to baseline levels. High levels of ERK expression have previously been shown to be associated with poor outcome in TNBC [41]. Contributing factors in this association may include ERK induction of IL-8 leading to increased migration and invasion and anchorage independent growth [42]. Indeed ERK inhibition in TNBC cells inhibits epithelial to mesenchymal transition, targets stem cell proliferation inhibits TNBC xenograft lung metastasis development in nude mouse models [43].

Furthermore, we show that the activation of the EGFR/ERK/MAPK pathway has a critical role in the development of a pro-inflammatory phenotype induced by DETA/NO. COX-2 has been reported to be overexpressed in breast cancer compared to normal breast tissue [44, 45]. Its main metabolite, prostaglandin E2 (PGE2) has been shown to promote migration, invasion, proliferation and angiogenesis while inhibiting apoptosis [46, 47]. In this regard, we show that the activation of EGFR and ERK, by DETA/NO drives COX-2 overexpression in the BL2 HCC1806. However, it had no effect in the BL1 MDA-MB-468 where we couldn't detect any basal or induced expression of COX-2. We had previously shown iNOS was associated with elevated tumor expression of IL-8 in ER-negative breast cancer and was inducible by DETA/NO in TNBC cell lines [14], and that iNOS inhibition decreased TNBC tumor xenograft IL-6 and IL-8 mRNA expression [23]. Conversely we didn't see any significant influence of DETA/NO treatment on IL-6 secretion in any of the cell lines tested. Potentially, the induction of IL-6 by NO, maybe dependent on other factors present in the tumor microenvironment but absent in an *in vitro* setting. We did however find that DETA/NO induced IL-8 secretion in both basal-like cell lines and similar to ERK phosphorylation, IL-8 induction in the BL2 HCC1806 was dependent on EGFR signaling.

Both IL-1β and TNFα are present in the tumor microenvironment. While in acute doses they can have cytotoxic effects [48], when present chronically, they have a strong pro-tumoral effect [49–51]. While increased NO levels didn't effect IL-1β or TNFα in MCF-10A or MDA-MB-468 cells, in the BL2 HCC1806, the activation of the EGFR pathway by NO significantly increased IL-1β and TNFα release, accompanied by TNFα dependent NF-κB activation. NF-κB regulates the expression of genes involved in key cellular processes such as adhesion, cell cycle, or apoptosis [52, 53]. These results highlights the cross-talk between EGFR signaling and inflammation processes that are critical in tumor metastasis in BL2 TNBC. The pro-inflammatory cytokine IL-8 has been previously shown to induce increased cancer stem cell renewal, cell invasion and metastasis and epithelial to mesenchymal transition [14, 54], and we now show a novel mechanism that that links high NO levels to EGFR-dependent IL-8 induction in BL2 cells.

As mentioned above, ERK activation is associated with increased migration and invasion [42]. Expression levels and activity of the MMPs are finely regulated during the tumor metastasis. We report DETA/NO increased expression of MMP1 and MMP9 mRNA and increased MMP9 enzymatic activity in the BL2 HCC1806, in an EGFR-dependent manner. Cell migration and invasion were regulated by NO through the EGFR/MAPK pathway, with NO significantly increasing cell migration in both TNBC cells, and in contrast inhibiting migration and invasion of normal MCF-10As. As we can't fully explain why the MCF-10A show reduced migration in the absence of a negative effect on MMP or IL-8 activity, further studies are needed to understand the biphasic effects of NO in tumoral versus non-tumoral breast cells. Additionally MCF-10A have their limitations as a normal control cell line, due to their immortalized status, however we believe that they are the best available control basal cell line, as primary mammary epithelial cells are ER positive. Indeed the MCF-10A exhibit higher rates of cell migration than either TNBC cell line, which reflects previous reports that they have greater colony formation potential than MDA-MB-468 or HCC1806 [55]. Similarly invasion through gelatin (MMP9 substrate) or collagen (MMP1 substrate) matrices was enhanced by DETA/NO, in BL2 but not in BL1 cells, reflecting results observed regarding MMPs expression and activity. However it should be noted that additional and/or more relevant mediators such as uPA/PAI-1 [56] or cathepsins [56, 57], may play a key role in the observed effects. These effects were totally or at least partially reverted when we combined the NO donor with the EGFR or MAPK inhibitors highlighting the importance of the EGFR/MAPK pathway regulating tumor progression in a pro-inflammatory environment.

To summarize, we examined the effect of high levels of NO in three different triple negative, basal like cell lines. Our data indicates that iNOS is a predictor of poor outcome in TNBC patients and particularly in basal-like patients. In deciphering the potential mechanism of action, we used the MCF-10A as a non-tumorigenic control cell line, and the MDA-MB-468 and the HCC1806 as representative examples of the BL1 and BL2 subtypes, and found the effects of DETA/NO to be more pronounced on the BL2 cell line. Lehman et al found that, while not statistically significant, patients with BL2 had a worse outcome than BL1 patients. We find that, in response to high levels of NO, the BL2 cell line (HCC1806) shows induction of pro-inflammatory signaling, NF-κB dependent gene-expression, increased migration and invasion which is dependent on EGFR signaling, an intrinsic feature of the BL2 subtype. In this way, high iNOS expression in the tumor samples may contribute to the poor outcome observed in basal-like breast cancer patients by enhanced pro-tumorigenic signaling in the BL2 subtype.

Despite EGFR being highly expressed in a large proportion of TNBC patients, the employment of engineered monoclonal antibodies (mABs) against EGFR is not approved for the treatment of breast cancer, while it is approved for other types of cancer such as colorectal cancer. In recent years, many research groups have pointed to the necessity of reconsidering this approach in TNBC, given the lack of an effective treatment [58–60]. As a consequence, two phase II clinical trials were developed, reporting non-statistically significant differences when combining conventional chemotherapeutic agents with EGFR inhibitor [61, 62]. However, no patient selection criteria were included in these trials, and considering the heterogeneity of the TNBC subtype this could be something to take into consideration delving in the need of stratifying TNBCs for future trials [63]. In this work, we aimed to shed light on the stratification of the TNBC. We provide substantial clinical and *in vitro* data showing that basal-like TNBC with high levels of expression of iNOS may be candidates for EGFR targeted therapeutics.

## MATERIALS AND METHODS

### Tissue collection and construction of tissue microarray

Paraffin-embedded (n = 209) tumor specimens were obtained from breast cancer patients diagnosed with TNBC (confirmed ER/PR/HER2 negative) at Galway University Hospitals between 1999-2016. Areas of tumor were identified by the pathologist and tissue microarray constructed. Clinical and pathological information were obtained from medical oncology and pathology reports. Disease staging was performed according to the tumor–node–metastasis (TNM) system of the American Joint Committee on Cancer/Union Internationale Contre le Cancer (AJCC/UICC). The Nottingham system was used to determine tumor grade. The collection of tumor specimens, and clinical and pathological information was reviewed and approved by the Galway University Hospitals Ethics Committee (Approval #CA1012). Recurrence free survival was defined as no recurrence at the local site (breast), regional (lymph nodes) or at distant sites. Metastasis free survival was defined as no metastasis to distant sites. Patients who had distant metastasis at diagnosis were excluded from recurrence free survival and metastasis free survival analysis. Breast cancer specific survival was defined as a cause of death due to breast cancer, and excluded death from other causes (non-breast cancer related).

### Immunohistochemistry

IHC was performed as previously described [64]. Briefly, protein expression in the TNBC samples was evaluated using a 1:200 dilution of mouse-monoclonal (clone 4E5) iNOS Antibody (MA5-17139 Invitrogen). The intensity of the staining received a score of 0–3 if the staining was negative, weak, moderate, or strong. The TMAs were scored by two pathologist and consensus reached.

### Cell culture

All the cell lines were purchased from the ATCC in 2014 (LGC Standards Ltd, Teddington, UK) and maintained at 37°C in a fully humidified 5%CO2 atmosphere. All cells were frozen down within 3 passages of receipt and subsequently used within a maximum of 10 passages. MCF-10A was grown in Dulbecco's Modified Eagle's Medium/Nutrient F-12 Ham (DMEM:F12) with 15mM HEPES buffer, 5% horse serum, EGF (10 μg/mL), hydrocortisone (0.5 μg/mL), insulin (10 μg/mL), cholera toxin (0.1 μg/mL), penicillin (100 units/mL) and streptomycin (0.1 mg/mL). MDA-MB-468 and HCC1806 were grown in RPMI-1640 with 10% fetal bovine serum and penicillin (100 IU/mL) and streptomycin (0.1 mg/mL). For experimental purposes, the cells were serum starved in RPMI-1640 media 4 hours before being treated. All the inhibitors employed for this study and their correspondent vehicles, PD153035 (100nM), PD198306 (200nM), Infliximab (5μg/ml) or 0.01% DMSO were administered, 60 minutes before treatment with 50μM NaOH (vehicle) or 0.1/0.3/0.5mM DETA/NO (Diethylenetriamine/nitric oxide adduct). Cells were rinsed twice with cold PBS and lysed directly on the dish with cold RIPA buffer (Sigma) supplemented with protease and phosphatases inhibitors, scraped, and spun at 14,000 g for 15 minutes at 4°C. Supernatant was collected and stored at −20°C for Western blot analysis of protein expression.

### Constructs and transfection techniques

The NF-кB Luciferase reporters driven by 5X wild type (5X NF-кB-Luc) (pNF-кB-Luc plasmid, Stratagene, Santa Clara, CA, USA) or negative control (no NF-кB binding sites-Luc) (pCIS-CK, Stratagene) were used in this study. MCF-10A, MDA-MB-468 and HCC1806 cell lines were plated in 6-well plates and transfected with 1μg of appropriate plasmid using Lipofectamine-LTX reagent (Thermo-Fisher) according to the manufacturer's instructions. Twenty-four hours post transfection, cells were treated with Infliximab for 1 hour followed by 50μM NaOH or 0.5mM DETA/NO. Twenty-four hours post treatment, cells were lysed and analyzed for luciferase activity.

### Western blot analysis

Protein concentrations were determined with the Bio-Rad Protein Assay. Western blot analysis was performed according to standard procedures, and 30–50μg of total protein was loaded per lane. The protein bands were visualized using the Super-Signal West Pico Chemiluminescent Substrate (Pierce). The following antibodies were used to detect proteins of interest: rabbit-polyclonal Phospho-EGF Receptor Y1068 (2234), rabbit-polyclonal Phospho-EGF Receptor Y1045 (2237), rabbit-monoclonal Phospho-EGF Receptor Y1173 (4407), mouse-monoclonal EGF Receptor (2239), rabbit-monoclonal Phospho-Erk1/2 T202/Y204 (4370), rabbit-monoclonal Erk1/2 (4695) all from Cell Signaling Technology, rabbit-monoclonal COX-2 (Thermo-Scientific. #RM-9121-S0) and mouse-monoclonal β-Actin (Sigma-Aldrich, A5441).

### Cytokine quantification

After treatment, cell culture supernatants were collected for cytokine quantification and spun at 180g for 5 minutes to remove debris, and stored at -80°C. These supernatants were analyzed with the Meso Scale Discovery (MSD) Human ProInflammatory Panel II (4-Plex) Tissue Culture Kit (K15025B). 25ul of sample per well were used to quantify the presence of IL-1β, IL-6, IL-8 and TNFα.

### Gelatin zymography

Activity MMP-2 and MMP-9 was measured by gelatin zymography. Briefly, the cell lines were seeded in 100mm petri dishes and treated with drugs in 10ml volume. After 24 hours the supernatants were collected and concentrated using Amicon Ultra-4 Centrifugal Filter Unit with Ultracel-30 membrane (Merck Millipore, UFC803024). Samples were electrophoresed on a 8% SDS-PAGE gel containing 0.2% gelatin. The gels were washed in 2.5% Triton X-100 three times and incubated overnight (20 mM TRIS, 5 mM CaCl2, pH 7.4) at 37°C. After staining with Commassie blue and destaining with a methanol/acetic acid mixture, gelatinolytic activity was identified as white bands over a blue background.

### Detection of matrix metalloproteinases gene expression

RT- PCR was performed as previously desc-ribed [65]. The following primers were used: 5′- AAAGGGAATAAGTACTGGGC-3′ MMP1 forward; 5′- CAGTGTTTTCCTCAGAAAGAG-3′ MMP1 reverse; 5′-GTGATCTTGACCAGAATACC-3′ MMP2 forward; 5′- GCCAATGATCCTGTATGTG-3′ MMP2 reverse; 5′- AAGGATGGGAAGTACTGG-3′ MMP9 forward; 5′-GCCCAGAGAAGAAGAAAAG -3′ MMP9 reverse; 5′-AGAGCTACGAGCTGCCTGAC-3′ β-actin forward; 5′- AGCACTGTGTTGGCGTACAG-3′ β-actin reverse. Thermal cycling 95°C for 2 min, followed by 40 cycles of 95°C for 5s and 60°C for 15s. Gene expression levels were calculated using the delta Ct method.

### Invasion and motility assays

Cells were harvested and resuspended in RPMI1640 containing 5% of FBS at a concentration of 1×10^6^ cells/ml. 100μl of the cell suspension and 100ul of DETA/NO and PD153035 or PD198306 at the appropriated concentrations were added to 8.0μm pore size inserts (Falcon), in a 24-well plate containing 0.5ml of RMPI1640 supplemented with 10% FBS. For the invasion assays, the inserts were previously coated with 1mg/ml of rat tail collagen (C7661 Sigma-Aldrich), or 0.2% of porcine skin gelatin (G1890 Sigma-Aldrich). Cells were incubated at 37°C for 24 hours for motility assays and 48 hours for invasion assays. After this time, the inner side of the insert was wiped with a wet cotton swab to remove the cells, while the outer side of the insert was gently rinsed with PBS and stained with 0.25% crystal violet for 5 minutes, rinsed again, and then allowed to dry. The inserts were then viewed under the microscope and the cells over a total of 5 random fields were counted at ×200 magnification, to determine the relative number of invading/migrating cells.

### Statistical analysis

Data analysis was performed using the Stata/SE 14 (Stata Corp, College Station, TX) and GraphPad Prism 5 (GraphPad Software, San Diego, CA) statistical software packages. All statistical tests were two-sided. P<0.05 was considered statistically significant. Survival was determined for the period from the date of hospital admission to the date of the last completed search for death entries on the Hospital system. Median and mean follow-up times for breast cancer survival were 46 months and 57 months, respectively (range: 1 to 186 months). A total of 58 of 209 patients died during this period. 9 patients who died of other causes were excluded from breast cancer specific survival. Kaplan–Meier curves and log-rank test for equality of the survival function was used for univariate survival analysis. Cell culture experimental data are represented as the mean±SEM of three independent biological replicates. Two-way ANOVA and Bonferroni tests were performed.

## SUPPLEMENTARY FIGURE



## Abbreviations

BL1: basal-like 1
BL2: basal-like 2
COX-2: cyclooxygenase-2
DETA/NO: Diethylenetriamine/nitric oxide adduct
EGFR: epidermal growth factor receptor
ER: estrogen receptors
IL: interleukin
iNOS: inducible nitric oxide synthase
LAR: luminal androgen receptor
MMP: matrix metalloproteinase
NF-κB: Nuclear factor-kappa B
NO: nitric oxide
PGE2: prostaglandin E2
PR: progesterone receptors
RTK: receptor tyrosine kinases
TNBC: triple negative breast cancer
TNFα: tumor necrosis factor alpha
