# Supplementary material for: Impact of inducible nitric oxide synthase (iNOS) expression on triple negative breast cancer outcome and activation of EGFR and ERK signaling pathways
Source: Oncotarget. 2017 Jul 26;8(46):80568–88. doi: 10.18632/oncotarget.19631 (PMC5655221; doi:10.18632/oncotarget.19631)
Supplement: Supplementary file 1 [file oncotarget-08-80568-s001.pdf]

Impact of inducible nitric oxide synthase (iNOS) expression on triple negative breast cancer outcome and activation of EGFR and ERK signaling pathways

SUPPLEMENTARY MATERIALS

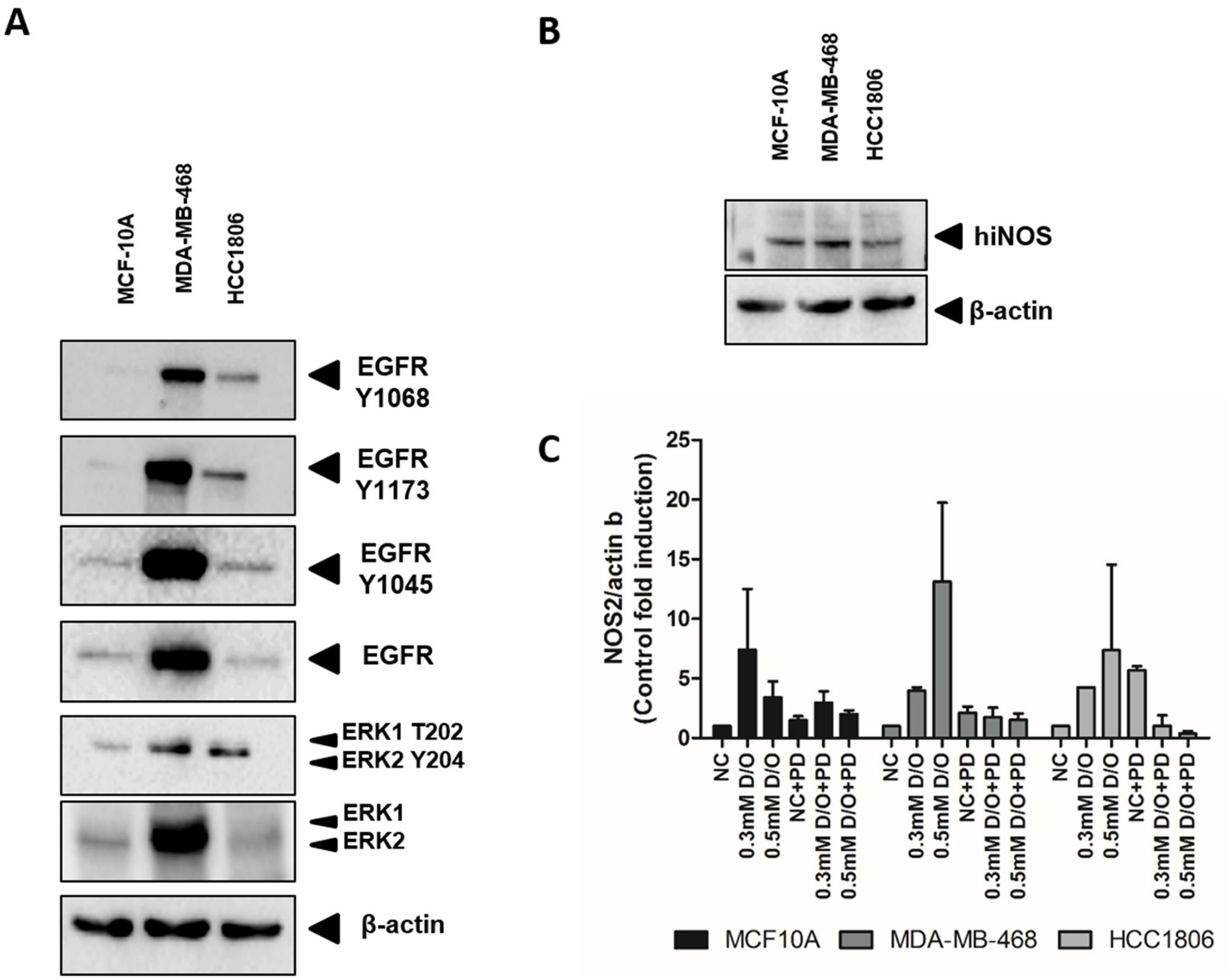

Supplementary Figure 1: Basal expression levels of **(A)** phospho-EGFR and phospho ERK and **(B)** iNOS in MCF-10A, MDA-MB-468 and HCC1806 cells. **(C)** Impact of DETA/NO +/- EGFR inhibition on iNOS mRNA expression in MCF-10A, MDA-MB-468 and HCC1806 cells.
